# Supplementary material for: Communication-efficient algorithms for solving pressure Poisson equation for multiphase flows using parallel computers
Source: PLoS One. 2022 Nov 22;17(11):e0277940. doi: 10.1371/journal.pone.0277940 (PMC9681093; doi:10.1371/journal.pone.0277940)
Supplement: Attachment — Submitted filename: Response to Reviewers.pdf [file pone.0277940.s001.pdf]

Response to the reviewers for paper titled "Communication-Efficient Algorithms for Solving Pressure Poisson Equation for Multiphase Flows using Parallel Computers"

Soumyadip Ghosh, Jiacai Lu, Vijay Gupta and Gretar Tryggvason

October 11, 2022

We would like to thank the editors and the reviewers for their insightful comments and input. We have changed the paper in response to their comments and believe that the paper has improved substantially as a result. We provide a summary of the response to each individual comment below. Please note that all references cited in this document are listed at the end of this response, and thus the numbering may not correspond to what is found in the paper. Further, all the changes in our revised manuscript are highlighted in blue, so that they can be easily identified. For your convenience, the comments of the editors and the reviewers are presented below in *italics*.

1 Response to the Editors

We thank the editors for handling the paper and for their comments. As suggested, we have addressed the comments of the reviewers in our revision.

2 Response to the Reviewers

We thank the reviewers for their expertise and assessment of our work. In the following, we address their concerns point by point.

2.1 Reviewer 1

- *Reading the authors' previous work, I feel that this work lacks novelty. So the first question I have for them is this: what is the difference between this work and your previous work conceptually?*

  We apologize for the lack of clarity regarding the difference between this work and our previous published works and address them here. The papers [1, 2] also describe an adaptive event-triggered communication algorithm, but for the specialized case of parallel stochastic gradient descent in machine learning. Our successive over-relaxation

PDE solver in this paper is conceptually quite different from the machine learning algorithm in [1, 2] due to the absence of random sampling in the former, hence the novelty in this work. The paper [3] introduces the idea of event-triggered communication in PDEs but it considers a constant threshold instead of the adaptive threshold proposed in this paper. Further [3] had experiments on a simple 2D domain from electrostatics which is different from the more sophisticated 3D domain from multiphase fluid flows considered in this paper. Finally, the previous work in [4] also considers a constant threshold instead of an adaptive threshold and does not provide the elaborate experimental verification as performed in this paper.

- *In the experimental results, authors said "This reduction in messages can not only lead to a decrease in simulation time as seen before, but also a decrease in energy consumption and congestion in interconnects." How did you measure energy consumption and congestion? What metrics did you use? Where are the curves that actually show that energy has been reduced ?*

  We thank the reviewer for pointing this out and added clarification after that sentence in the paper. Note that it is difficult to measure the reduction in energy consumption directly but we point to a metric in [5] which states that around 1-3 pJ is spent in moving 1 bit of data between processors connected in a network. This is one of the factors that motivated our work. We also recently came to know about Performance Application Programming Interface (PAPI) which is a toolkit with performance counters to measure energy/power - `https://icl.utk.edu/exa-papi/`. We would like to explore PAPI in our future work to see if it can measure exact energy savings for the communication. For congestion reduction, we refer to [6] which highlights the aspect of decrease in congestion with reduction in messages. Exact measurements for congestion reduction using our algorithms also requires detailed monitoring of the interconnect traffic which we leave for future work.

- *In the experimental results, authors said :"The effect of the decay d and horizon h on the total simulation time is shown in Fig 7." How many processing elements did you use to get these results? This information was not specified.*

  We included a note after this sentence, reminding the readers that the experimental results are based on the parameters mentioned in Table 1 earlier in the paper. To answer the question specifically, we used 200 PEs.

- *What are the complexity of your algorithms with regard to total execution time and number of communication rounds ? What is the different between the complexity of this work and your previous works?*

  For both the asynchronous and event-triggered algorithm, MPI one-sided communication adds stochasticity, so it is difficult to quantify their complexity. Further, for the event-triggered algorithm, the threshold for communication is adaptive, so the number of events vary across different boundaries and processors. We believe that these reasons make it intractable to establish theoretical algorithmic complexity. For the current paper, we have focused on experimental results of the algorithms and leave any possible theoretical complexity results to future work.

- *In section "Proposed Event-Triggered Communication Solver", authors said :"When this norm changes from the norm at previous communication by more than a specified threshold, the boundary values are communicated to the PE that is the intended receiver." Can there be interlocking situations? And what happens during a collective communication?*

  We appreciate this question and clarify it here. By interlocking situations, we assume that the reviewer means situations when communication is triggered at 2 PEs to each other. That situation can arise and will not lead to deadlock for the event-triggered solver. This is because MPI one-sided communication (MPI_Put) is used. To explain this, lets consider that the two PEs involved are PE0 and PE1 and both of them have an event of communication triggered simultaneously to each other. In that case, PE0 can just use MPI_Put to directly access the remote memory location in PE1 and write the message there without PE1's involvement. Similarly PE1 can do the same to PE0 independently. This is an advantage of one-sided communication over two-sided communication.

  The event-triggered communication algorithm as well as the asynchronous algorithm does not have any collective communication. The usual MPI_Allreduce present for convergence detection in the synchronous algorithm is replaced by a distributed convergence detection algorithm in our work. Further details are specified in the 2nd paragraph of the "Proposed Asynchronous Solver" section.

- *In section "Proposed Asynchronous Solver", authors said : "We note that other researchers [30] also developed asynchronous solvers with MPI one-sided communication for domain decomposition but in the context of restricted additive Schwarz solvers which is different from the SOR solver we consider". Can you clearly show us the difference ?*

  We are sorry for the lack of clarity and address it here. The paper by Nayak et al. [7] develops asynchronous restricted additive Schwarz solvers and conducts experiments with them on GPUs. Schwarz solvers are two-level domain decomposition solvers where the inner solver is often a Krylov subspace solver like conjugate gradient or GMRES and the outer solver is Block Jacobi [8]. In contrast, this paper does not consider a Schwarz solver but the simpler one-level SOR solver. Thus the SOR algorithm is conceptually different from the Schwarz solvers' algorithm. We added a sentence towards the end of 1st paragraph in Section 4 regarding this.

- *In the introduction, authors said : "However, these s-step methods considered parallelization using operator decomposition which is different from the parallelization using domain decomposition considered in this paper." Can you clearly show us the difference ?*

  We apologize for the absence of clarity. The s-step methods studied in literature consider parallelization of matrix-matrix and matrix-vector multiplications. In other words, the matrix and/or associated vectors are decomposed in a row-striped manner and each partition is assigned to a PE. This is what we meant by operator decomposition. Thus the partition assigned to each PE doesn't really correspond to a decompo-

sition along the domain. In contrast, we consider domain decomposition wherein the grid points in the simulation domain are divided amongst the multiple PEs. Within each decomposed domain assigned to a PE, there is no operator decomposition, i.e., all matrix-matrix or matrix-vector multiplications in that domain are executed by just that PE. We added a sentence at the end of paragraph 3 in Section 1 in the paper to highlight this point.

We have also incorporated the suggestions proposed by the reviewer, including correcting the sentence construction mistakes.

2.2 Reviewer 2

- *The problem to be analyzed is not sufficiently specified, making it impossible to understand what exactly gets done. More precisely, the authors need to state the exact version of the SOR algorithm, the form of the coefficients rho in the figure from Figure 1, the number of iterations, overlap/data dependency resolution in terms of the classical SOR algorithm in terms of the domain decomposition, and similar settings. Note that I tried to compile and run the code from the given repository, but the instructions are insufficient. I managed to figure out the command line arguments from the source code, but there is apparently need for some input arguments that are hardcoded to the author's computer, rather than available from the repository. The repository needs to contain a state the works on a third-party computer, at least for such a simple case with no NDA issues.*

  We thank the reviewer for pointing this out. We have now added more details to the algorithm, such as the parameters of the SOR algorithm and the density ratios of the fluids. Also, the github repository has been updated with a detailed Readme and link to some sample coefficient matrices. We verified that the code now works on a third party computer after changing the file paths. Note that the computer needs to have MPI installed.

- *Regarding the asynchronous algorithm of Section 4, I would like the authors to state how precisely the neighboring information is used for the local computations: In general, we need to assume that the neighbor might be in the process of writing to the place where the receiver is just reading from. Is there a way that invalid data could appear, and is this operation guaranteed to be using valid data? I believe yes, as the data will not get written in bit granularity, but cacheline granularity, and at the remaining gap cache coherency will ensure that anything that has arrived will be the long-term valid state.*

  We have added details about the MPI one-sided communication in Section 4 paragraph 2. As already specified there, we define a public memory *window* and use MPI_Put with passive target synchronization using MPI_Win_lock/unlock to do the neighbor communication. Once MPI_Win_unlock is called, the sender has to finish the communication, so the data is now available for the receiver to do local computations. Before the MPI_Win_unlock is called, there is no guarantee that the sender has finished writing the data to the window. If the receiver reads the data from the window at this time, some cells in the window might not be updated with the

latest data - so the values in the window may not correspond to the same iteration. In order to prevent this phenomenon of inconsistent data, we copy the data from the window to another array after the MPI_Win_unlock and use values from this array for the local computations. This will ensure that consistent data from a particular iteration is being used. See these lines in the code where the copy is being done - `https://github.com/soumyadipghosh/eventpde/blob/main/event.c#L482` and `https://github.com/soumyadipghosh/eventpde/blob/main/event.c#L559`

- *The overall motivation for choosing the simple algorithm SOR needs to be reframed. If I understand the results in Figures 5 and 9 correctly, the iteration count is between 100k and 1 million. For solving a Poisson problem, this is not competitive, and findings may be skewed due to this selection, see also below. While the authors mention this in the introduction (page 3, first full paragraph), the point is that even variable-coefficient Poisson problems can be solved at the equivalent of with 10-100 applications of the fine-level operator. This is achievable with multigrid methods, taking SOR smoothening and some 5-10 iterations. Similar costs can be obtained with BDDC or FETI-DP methods. To be specific, I believe this problem can be solved in around 1 second for 48 cores (1 node) of the given AMD architecture, which need to be compared to around 1700 seconds on 200 processing elements (cores?) according to Table 2. Obviously, 1 second versus 1700 seconds sets very different requirements on the communication timings, and this needs to be set in proper context. The authors mention hypre as a frequently used solver: I strongly suggest to add its timings; the objective will not be to beat it or even be close in timings (as hypre should be near optimal), it is needed to set a relevant anchor. Note that I do see value in keeping this simpler algorithm (as a first research step to gain insight), but the conclusions need nonetheless be anchored against the current state of the art.*

  Our selection of SOR is motivated by our desire to focus on the communication aspect and not the specifics of the solver. We did, in particular, select the parameters of the problem (the density ratio to be very high) such that the solver would require a large number of iterations to converge. This detail has been added to the end of last paragraph in Section 2. We are fully aware that today SOR is not a competitive solver and that it is particularly slow for a large and discontinuous change in the coefficients. However, the need for a large number of iterations requires extensive communications and we feel that provides a good testbed for the ideas explored in the manuscript. Our emphasis is not on how the SOR solver compares with other methods, but how much speedup is achieved for the same solver with our strategy. This is now emphasized in the revised manuscript, where we stress that the specific savings would be different for different problems and different solvers.

- *Regarding the previous comment, I definitely see value of the proposed ingredients for multigrid smoothers, so at least section 4 seems to be very relevant for a moderate number of sweeps, say 5-10, given the right background. However, the event-based solver is questionable at the very least: The authors argue that the changes of boundary data gets less as the SOR iteration is advanced, advocating some extrapolation at later stages. If I interpret the results correctly, this is because the SOR iteration smoothens*

the error, leaving only low-frequency parts of the error, where the nearest-neighbor exchange is now between very similar values. However, that would be the place where one really needs to go to coarser grids (or a BDDC coarse space), because the work spent on a fine grid is really in vein: A lot of work, but extremely slow convergence. Without proper background and additional motivation, I cannot see how this would be useful for a practically relevant PDE solver, apart from educational purposes. Again, I emphasize that I do not dispute the value of event-based steps in general, but here the events are applied to a property that a state-of-the-art algorithm will not expose, because it has employed much more efficient steps.

We agree with the reviewer. There are many ways to accelerate the solutions using more advanced solvers and adaptive grids. However, here we are focusing just on the communications. We feel that in order to isolate those, it is important to keep the solver as simple as possible.

- *Coming to the quality of the results, the first data point given in Table 2 suggests a run time of 10,346 seconds for the synchronous solver and 1,691 seconds for the proposed solver, which the authors credit to the one-sided MPI communication. In my experience, the difference is way too high, and I would rather expect the advantage to be in the range of 5-10% in the best case [*]. Since I am not able to run the authors' code, my impression is that something in the experiment is broken. To gain understanding, it would be important to list the number of iterations in the synchronous case and compare that number to some relevant statistical average of the number of local iterations across all 200 PEs in the asynchronous case. If these two numbers different substantially, the one-sided MPI communication is not the cause, and something similar could then also be emulated with MPI_Send/Recv. Or put differently, if the iteration count explains the difference, the finding is useless and linked to a bad algorithm choice. Also beyond the question of iteration counts, to make a valuable scientific contribution the authors would need to explain the origin of the difference in timings, by identifying a bottleneck in the synchronous algorithm (in the sense of timings of a single iteration, and comparing the achieved throughput to the hardware capabilities), and showing how the one-sided MPI algorithm relaxes on this bottleneck. The authors vaguely talk about the cost of exchanging messages, but I am not convinced. This can be seen from timings: If we assume the synchronous solver runs 10k seconds and takes* 10^6 *(one million) iterations, the time per iteration is 0.01 seconds. Regular MPI ghost exchange scales well all the way to 1e-4 seconds on architecture similar to the one used here [**], so there must be something else, e.g. improper load balancing or other aspects.*

  We apologize for the lack of clarity with respect to the results. The difference between the synchronous and asynchronous algorithm is not just the replacement of two-sided with one-sided communication but also the removal of collective communication for convergence detection. The MPI_AllReduce collective operation is used in the synchronous algorithm to calculate the global residual that determines convergence. This requires more time than ghost cell communication. Further the time required for MPI_AllReduce increases with the number of PEs involved in the simulation unlike ghost cell communication. In the asynchronous algorithm, there is no collective com-

munication and the convergence detection is done in a distributed manner as specified in Algorithm C. Therefore the time taken for MPI_AllReduce for the number of iterations in the synchronous solver is saved. Hence we see the 6x performance improvement with the asynchronous solver. We have added a sentence at the end of the last paragraph in Section 4 to highlight this point.

- *The authors state that they use 200 processing elements (PEs). I assume this means 200 MPI ranks. As the machine they use has nodes with 48 cores each, this means that some nodes are not fully populated. The exact mapping of work to cores and nodes needs to be specified, also regarding the possible load balancing problem. Generally, I strongly recommend to use a number of subdomains that completely fill a node. Also, to give a proper analysis of the algorithms, experiments on different numbers of PEs (nodes) would be very helpful, in the context of a strong scaling experiment.*

  Thanks for highlighting this aspect, we forgot to mention something here. Even though the nodes have 48 cores, we used only 40 cores in each to ensure that all the nodes are equally populated. In other words, we used the following MPI run command: `mpirun -N 40 ...` where `-N` is the OpenMPI option for specifying the number of processes per node. So with 40 processes per node, 5 nodes were reserved in order to execute the simulations with 200 PEs. We added a sentence to the last paragraph of Section 2 regarding this fact. Note that we had to choose 200 PEs for the simulations because of the discretization of the grid we had access to.

- *The one-sided communication adds a statistical component to the algorithm and its performance. This would demand for a statistical analysis of the results: If I execute the algorithm 10 times, I would expect different results, both in terms of iterations to convergence (here, iteration needs to be an average on its own as different subdomain will produce different numbers) and timings. The result needs to be shown with its statistical distribution, error bars around some expectations, and a proper discussion needs to be added.*

  The reviewer is correct, thank you for highlighting this. Indeed we had used an average over 3 runs for each data point in Figs 7, 8 and 9. We added a sentence to mention this at the beginning of the 2nd paragraph in Section 6. We believe that including the error bars for each data point in the plots will fill it with clutter which might hamper their readability.

- *The presentation quality is rather poor overall, e.g. Figure 5 should use logarithmic scaling of the x axis, maybe also Figure 6. This is one example, other figures should also be looked at to provide a good presentation.*

  Our apologies for the presentation quality, we took steps to address that. Fig 5 and 6 now have logarithmic scaling of the x axes.

- *I suggest the authors to tone down or skip their high-level discussion about HPC, microprocessors or parallelism in the introduction and conclusions. I feel that the authors do not really know what they are talking about, highlighting some tangentially relevant metrics they found in some "keynote" papers, but completely ignoring more mundane*

and more relevant problems with their algorithms and presentation. Without a sense for the absolute timings and bottlenecks, the drawn conclusions might or might not be what is relevant for state-of-the-art solvers. Note that I do not dispute the study of simplified problems or non-optimal algorithms for the sake of exploring fundamentally new ingredients, but it needs to be done against a proper understanding of the state-of-the-art with relevant links.

We apologize for the high level discussion, we have removed it now. We just provide some context behind the motivation for this work and then summarize the findings in the paper in this section.

References

[1] Soumyadip Ghosh, Bernardo Aquino, and Vijay Gupta. Eventgrad: Event-triggered communication in parallel machine learning. *Neurocomputing*, 483:474–487, 2022.

[2] Soumyadip Ghosh and Vijay Gupta. Eventgrad: Event-triggered communication in parallel stochastic gradient descent. In *2020 IEEE/ACM Workshop on Machine Learning in High Performance Computing Environments (MLHPC) and Workshop on Artificial Intelligence and Machine Learning for Scientific Applications (AI4S)*, pages 1–8. IEEE, 2020.

[3] Soumyadip Ghosh, Kamal K. Saha, Vijay Gupta, and Gretar Tryggvason. Event-triggered communication in parallel computing. In *2018 IEEE/ACM 9th Workshop on Latest Advances in Scalable Algorithms for Large-Scale Systems (scalA)*, pages 1–8. IEEE, 2018.

[4] Soumyadip Ghosh, Kamal K Saha, Vijay Gupta, and Gretar Tryggvason. Parallel computation using event-triggered communication. In *2019 American Control Conference (ACC)*, pages 4000–4005. IEEE, 2019.

[5] Keren Bergman, Shekhar Borkar, Dan Campbell, William Carlson, William Dally, Monty Denneau, Paul Franzon, William Harrod, Jon Hiller, Sherman Karp, Stephen Keckler, Dean Klein, Robert Lucas, Mark Richards, Al Scarpelli, Steven Scott, Allan Snavely, Thomas Sterling, R. Stanley Williams, Katherine Yelick, Keren Bergman, Shekhar Borkar, Dan Campbell, William Carlson, William Dally, Monty Denneau, Paul Franzon, William Harrod, Jon Hiller, Stephen Keckler, Dean Klein, Peter Kogge, R. Stanley Williams, and Katherine Yelick. Exascale computing study: Technology challenges in achieving exascale systems Peter Kogge, Editor & Study lead. *Defense Advanced Research Projects Agency Information Processing Techniques Office (DARPA IPTO), Tech. Rep*, 15, 2008.

[6] Torsten Hoefler, Timo Schneider, and Andrew Lumsdaine. Multistage switches are not crossbars: Effects of static routing in high-performance networks. In *2008 IEEE International Conference on Cluster Computing*, pages 116–125. IEEE, 2008.

[7] Pratik Nayak, Terry Cojean, and Hartwig Anzt. Evaluating asynchronous schwarz solvers on gpus. *The International Journal of High Performance Computing Applications*, 35(3):226–236, 2021.

[8] Barry Smith, Petter Bjorstad, and William Gropp. *Domain Decomposition: Parallel Multilevel Methods for Elliptic Partial Differential Equations.* Cambridge University Press, 2004.
